# The role of tryptophan derivatives as anti-kinetoplastid agents

**DOI:** 10.1016/j.heliyon.2023.e23895

**Published:** 2023-12-15

**Authors:** Ewura-Esi Manful, Aboagye Kwarteng Dofuor, Theresa Manful Gwira

**Affiliations:** a Division of Molecular Biology and Human Genetics, Stellenbosch University, South Africa; b Department of Biological Sciences, University of Environment and Sustainable Development, Somanya, Ghana; c West African Center for Cell Biology of Infectious Pathogens, University of Ghana, Legon, Ghana; d Department of Biochemistry, Cell and Molecular Biology, University of Ghana, Legon, Ghana

**Keywords:** Tryptophan derivatives, Chemotherapy, Anti-Kinetoplastids, Trypanosomiasis, Leishmaniasis, Chagas disease

## Abstract

Kinetoplastids are the causative agents for a spectrum of vector-borne diseases including Leishmaniasis, Chagas disease and Trypanosomiasis that affect millions of people worldwide. In the absence of safe and effective vaccines, chemotherapy, in conjunction with vector control, remain the most significant control approach for kinetoplastid diseases. However, commercially available treatment for these neglected tropical diseases frequently ends up with toxic side effects and increasing resistance. To meet the rising need for innovative medications, alternative chemotherapeutic agents are required. Moreover, insights into target-based mode of action of chemotherapeutic agents are required if novel drugs that may outwit resistance to commercially available drugs are to be developed. Tryptophan has been implicated in a variety of diseases and disorders due to its fundamental role as a precursor to several bioactive metabolites, as well as its importance in the improvement of health and nutrition, diagnostics, and therapeutics. The regulation of tryptophan metabolism plays a fundamental role in the growth of kinetoplastids. Moreover, the levels of tryptophan may serve as a biomarker to distinguish between the stages of kinetoplastids making it an important amino acid to explore for drug targets. The main aim of this review is thus to provide a comprehensive literature synthesis of tryptophan derivatives to explore as potential anti-kinetoplastids. Here we highlight the role of tryptophan derivatives as chemotherapeutic agents against kinetoplastids. The reviewed compounds provide insights into potential new drug interventions that may combat the increasing problem of anti-kinetoplastid resistance.

## 1 Introduction

High rates of morbidity and death, particularly in developing nations, are caused by kinetoplastid parasites leading to neglected tropical diseases (NTDs), which include leishmaniasis (cutaneous, visceral, and mucocutaneous), Chagas disease (American Trypanosomiasis), and sleeping sickness (Human African Trypanosomiasis) (HAT), as typically caused by *Leishmania* spp, *Trypanosoma cruzi*, and *Trypanosoma brucei*, respectively [1]. The vectors that transmit these parasites into the hosts are blood sucking insects such as tsetse fly for *Trypanosoma* spp [2] and sandflies for *Leishmania* spp [3]. Low-income nations and disadvantaged groups are increasingly at risk of infection, resulting in high mortality and morbidity, as well as a significant economic toll. Kinetoplastid infections, afflict about billion individuals and have been documented in roughly 20 million cases globally, leading to over 95,000 fatalities each year [4]. These infections thus pose serious health and financial risks, especially in locations where they are prevalent, amid the coordinated efforts to eradicate them.

Despite the efforts, existing treatment regimen for NTDs have drawbacks and limitations. For instance, pentamidine and eflornithine, which are the current drugs available for HAT have been largely associated with major side effects. In addition they show varied effects at different stages of the infection, as well as efficacy variability against different subspecies of *T. brucei* [5]. Benznidazole and nifurtimox, the current recommended drugs for treating Chagas disease also have long term regimen with many side effects that jeopardize the effectiveness of the drugs. Additionally, the efficacy of these drugs may decrease over the course of an infection despite being effective in the early stages [6]. Miltefosine, pentavalent antimonial, amphotericin B deoxycholate and pentamidine that are currently employed for leishmaniasis have drawbacks and side effects [7]. Current treatments for kinetoplastids are mostly insufficient, ineffective, and extremely toxic with a growing evidence of drug resistance [8].

Given the absence of safe and effective vaccines, the primary approach for controlling kinetoplastid diseases continues to be chemotherapy combined with vector control. Nevertheless, existing commercial treatments for these neglected tropical diseases often lead to toxic side effects and an increase in drug resistance. As a result, there is the need for more alternative drugs with greater efficacy and effectiveness, short term regimen and less side effects. Meeting the growing demand for effective medications requires the exploration of alternative chemotherapeutic agents. Furthermore, a comprehensive understanding of the target-based mechanisms of action of these agents is crucial for the development of new drugs that can overcome resistance to existing treatments.

Currently, natural compounds are receiving more attention for treating parasitic infections. Natural products provide an alternative supply of unexplored biologically active substances that might be employed as precursors for the production of novel therapeutic agents against parasites [8]. Recent efforts to decipher the structural and biological characteristics of natural products with anti-kinetoplastid activity have yielded molecules with significant therapeutic potential against kinetoplastids [8]. Numerous plants have been reported to have anti-kinetoplastid properties in various parts of the world [[9], [10], [11]].

Indole moieties, such as those present in the tryptophan (Trp) amino acid, can also be found in a wide range of naturally occurring compounds with different physiological characteristics [12]. Chemical alterations of amino acids with indole moieties have attracted the interest of researchers to develop novel compounds that may have a therapeutic impact against a variety of diseases. These compounds and their derivatives thus have the potential to serve as a source of pharmaceutically active drugs.

Trp, a vital amino acid, which contains an indole moiety has been implicated in a range of diseases and disorders due to its critical role as a precursor to various bioactive metabolites. Its significance extends to improving health, nutrition, diagnostics, and therapeutics. The regulation of Trp metabolism plays a pivotal role in the growth of kinetoplastids. Moreover, the levels of Trp can potentially serve as a biomarker for distinguishing between different stages of kinetoplastid development, making it an important amino acid to explore as a potential drug target. Since Trp plays fundamental roles in the regulation of the growth of kinetoplastids [13], its derivatives may be explored in the quest for novel anti-kinetoplastids. However, few studies have explored the potential role of Trp as an anti-kinetoplastid agent.

In this review, we highlight the therapeutic potential of Trp and its metabolites, the biosynthesis of Trp derivatives, experimental investigations on the effect of Trp derivatives against kinetoplastids growth and their future prospects as potential anti-kinetoplastids.

## 2 Methodology

This review was based upon a number of previously reported research work carried out on the structure and physiological roles of Trp in the light of potential anti-trypanosomatid activities of plant species. A literature review was conducted using comprehensive databases such as PubMed, PubChem, DrugBank, protein data bank (PDB) NCBI, Gene Ontology, UniProt, Prota4u and String Database.

## 3 Nature and lifestyle of kinetoplastids

Kinetoplastids are flagellated protozoans that have a DNA-containing region in their single big mitochondrion, referred to as a “kinetoplast” and this is what distinguishes them from other protozoans. Although the diverse kinetoplastid pathogens have comparable genetic architecture, and cellular structures, and go through morphological changes throughout their life cycles, these flagellated protozoans cause separate human illnesses and are transmitted by different insect vectors [14]. They are divided into two monophyletic groupings based upon their morphological characteristics, namely biflagellate bodonids and uniflagellate trypanosomatids. Some members of this class (kinetoplastida) have been proven to exist as free-living flagellates (for example, Bodo), whilst others are plant parasites (e.g., Phytomonas). Free-living kinetoplastids graze on bacteria in both marine and terrestrial settings. Kinetoplastids are known to be the etiologic agents of about three human diseases recognized as neglected tropical diseases (NTDs) by the World Health Organization. They include human African Trypanosomiasis (HAT) also known as African sleeping sickness, which is caused by two of the three subspecies of *Trypanosoma brucei*; Chagas disease, caused by *Trypanosoma cruzi,* and Leishmaniasis caused by various species of *Leishmania*.

The general size of the kinetoplast structure varies from species to species. In *T. brucei*, the kinetoplast is about 0.6 μm in diameter; in *T. cruzi,* it is around 1 μ m in diameter. This feature is different from the nucleus and has been found to contain “kDNA”, a DNA localized in the mitochondria [15]. Trypanosomatid kinetoplast DNA (kDNA) is a network of interconnected circular molecules divided into two types: maxicircles (20–40 kb) and minicircles (0.5–10 kb). Some researchers believe that kinetoplasts may contribute to *T. cruzi's* pathogenicity and that the minicircles they produce can integrate into the host genome to cause autoimmune reactions [16]. In addition, maxicircle gene deletions have also been linked to asymptomatic Chagas disease patients. The sequences of the few maxi circles are comparable, and they represent conventional mitochondrial genes. The thousands of minicircles are open-conformation circles (not supercoiled) that are interconnected with three neighbors to form a continuous network. Each minicircle encodes 1–4 short guide RNAs (gRNAs) with hundreds of distinct sequence types per cell, depending on the taxon [17]. The kinetoplast tends to move around within the cell during the trypanosome life cycle, although it always stays near the basal body [15]. During trypanosome life cycle, trypomastigotes, which is the flagellated form of trypanosomes transform into intracellular amastigotes after infecting cells from a variety of tissues. The kinetoplast is situated anterior to the nucleus in amastigotes, but posterior to the nucleus in trypomastigotes. The kinetoplast is usually found at the base of the flagella and was previously known to be somehow associated with the movement of the cell [15].

The life cycle of pathogenic kinetoplastids involves a variety of mammalian species, as well as insects, which are responsible for the majority of mammalian transmission [18]. As a result, the life cycles include the development of infection and survival inside different hosts - invertebrate and vertebrate hosts, as opposed to free-living kinetoplastids protozoans, as well as plant parasite kinetoplastids. Kinetoplastids exhibit a variety of morphological forms. These diverse physical features are connected with various life cycle phases in different species. Different morphological forms can be distinguished at each stage of an organism's life cycle, depending on the organism.

**Fig. 1:**
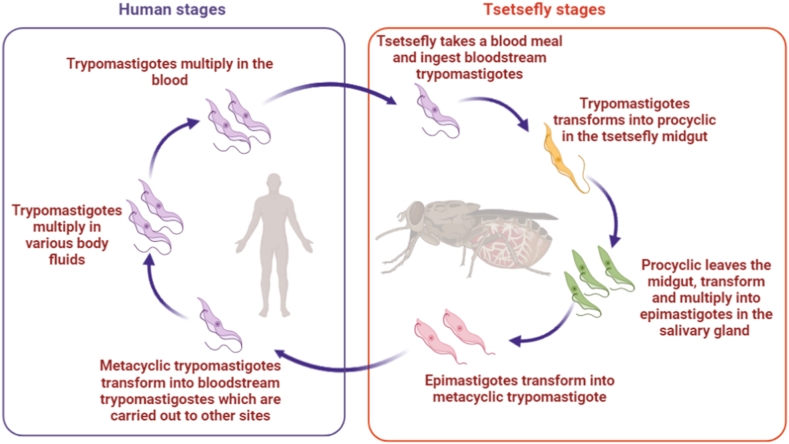
Life cycle of *T. brucei* - An infected tsetse fly injects metacyclic trypomastigotes into skin tissue while feeding on the mammalian host's blood. They transform into bloodstream trypomastigotes while inside the host, move to other parts of the body, infiltrate the blood, and continue replication. When a tsetse fly feeds on an infected mammalian host, it becomes infected with bloodstream trypomastigotes. The parasites evolve into procyclic trypomastigotes in the fly's midgut, proliferate by binary fission, exit the midgut, and consequently develop into epimastigotes. When the epimastigotes get to the fly's salivary glands, binary fission is used to continue their replication and later transform into metacyclic trypomastigotes.

*T. brucei* is spread by tsetse flies that feed on blood (Fig. 1). During a blood meal on an infected mammal, the tsetse picks up *T. brucei* bloodstream forms (BSF). Based upon morphological, biochemical, and biological features, two kinds of BSF may be distinguished: the long-slender BSF and the stumpy BSF. Long-slender BSFs are replicative, and they are responsible for parasitemia in the mammalian host for a long time. Long-slender BSF differentiates into non-replicative stumpy forms when they reach a particular population density, which is detected by a secreted “stumpy induction factor” (SIF). PCF multiplies and begins to migrate to the salivary gland. They undergo development into procyclic epimastigotes throughout this life cycle, which is finished once they reach the salivary gland. They eventually develop into infective, non-dividing metacyclic forms after colonizing the salivary gland, which will be implanted in a new mammalian host during the insect's next blood meal. To reproduce in the circulation and establish the infection, metacyclic develops into long-slender BSF. BSF can penetrate the blood-brain barrier and reach the central nervous system in the long run.

**Fig. 2:**
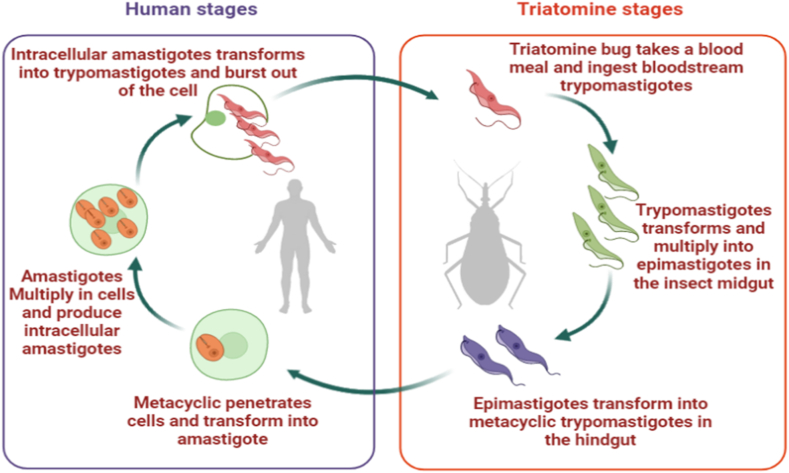
Life cycle of *T. cruzi*- An infected triatomine insect vector excretes trypomastigotes close to the site of a wound after a blood meal. Trypomastigotes enter the host through the wound. The trypomastigotes infiltrate cells close to the injection site within the host, where they transform into intracellular amastigotes. The amastigotes multiply and are releases into the bloodstream as trypomastigotes where they infect other cells and changes into intracellular amastigotes in the new cells. Bloodstream trypomastigotes do not multiply (unlike the bloodstream forms of African trypanosomes). When a triatomine feeds on an infected human blood, it ingests trypomastigotes which transform into epimastigotes in the insect's midgut. The parasites proliferate and differentiate in the midgut before transforming into infective metacyclic trypomastigotes in the hindgut.

*T. cruzi* is transmitted by triatomines that carry the infective forms of the parasites through their feaces. There are several modes of transmission, but oral transmission is one of the earliest and most critical for the parasite's zoonotic cycle. Many animals have been shown to become infected with *T. cruzi* when fed triatomines or other animals. Studies have shown that humans are infected by triatomine insect bites or contaminated food and water - food, fruits and water infected with T. cruzi from triatomine feces or reservoirs secretions [19]. *T. cruzi* transforms into epimastigotes that develop into infectious metacyclic forms. The parasite enters the circulation of mammals by mucosal membranes or a skin bite. They may infect a variety of mammalian cell types and develop into replicative amastigotes with a shortened flagellum. The parasites will then mature into trypomastigotes, which will burst the host cells and enter the circulation, where they will infect more cells or be taken up by the insect vector (Fig. 2) [3].

**Fig. 3:**
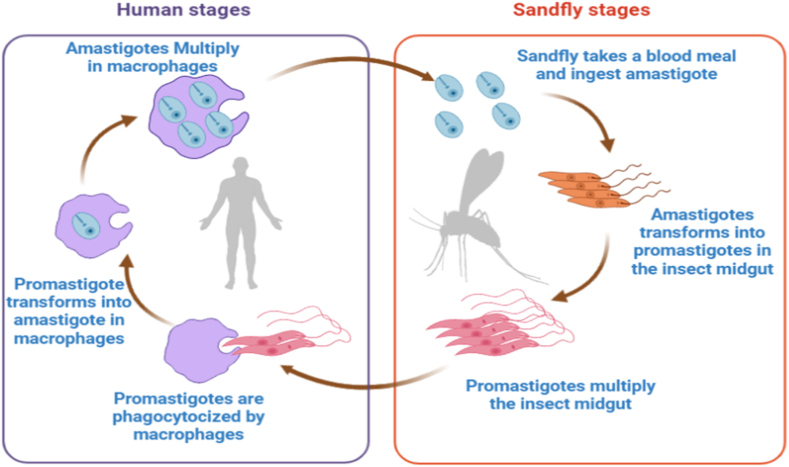
Life cycle of *Leishmania spp-* Phlebotomine sandfly females bite humans and transmit leishmaniasis. During a blood meal, the infected phlebotomine female sand fly finds a suitable host and injects the promastigote parasites into the skin. When these parasitic promastigotes enter the wound, they are phagocytized or consumed by macrophage cells. Macrophages phagocytize these promastigotes, and they differentiate into amastigotes. Amastigotes proliferate in infected cells and infect other phagocytic cells. A sandfly later becomes infected with the parasite when it consumes these infected cells while consuming blood from an infected human. The amastigotes grow inside the midgut of the sandflies and transform into promastigotes where they proliferate and go to the proboscis.

*Leishmania* has a two-stage life cycle in which they alternate between the extracellular promastigote stage in the sandfly vector and the intracellular amastigote stage in mammalian host macrophage phagolysosomes [20]. *Leishmania* spp. is spread by female phlebotomine sandflies that get infected after feeding on an infected animal (Fig. 3). Phlebotomine consumes infected macrophages, which are destroyed by hydrolytic enzymes found in the insect digestive tube during the bloodmeal. The amastigotes that are discharged differentiate into proliferative promastigotes that begin to multiply in the midgut. When nutrients are depleted and the pH is low, promastigotes develop into non-replicative, infective metacyclic promastigotes, which mostly concentrate in the stomodeal valve. These metacyclic forms will be injected into the mammalian host during the subsequent blood meal. Sandfly saliva components can act as attractors for host macrophages, which move to the bite site and phagocytize the *Leishmania* parasites. Metacyclic promastigotes can develop into replicative amastigotes once within the parasitophorous vacuole. Infected cells of *Leishmania* spp. can be ingested by a (non-infected) sandfly, which can then transmit it to a new mammalian host [20].

Several aspects of cellular biology are shared between the various kinetoplastids. Their flagella emerge from a pocket in the cell membrane where endocytosis also occurs. Their peroxisomes have been modified to undergo glycolysis and are so referred to as glycosomes. Moreover, the cell membrane is coated with a microtubule film and adorned with species-specific chemicals essential to their survival [14]. Also, sexual recombination has been demonstrated for *T. brucei*, predicted for *T. cruzi*, and may possibly occur in some *Leishmania* species. Furthermore, they divide through binary fission, which does not result in membrane disintegration or chromosomal condensation in the nucleus [14].

## 4 Sources and biological functions of tryptophan

In the early 1900s, Trp was discovered by an English chemist called Sir Frederick Gowland Hopkins from a casein protein [21]. Following its discovery, the structure of Trp was later elucidated in the year 1908 [22]. It is one of the nine essential amino acids for humans [23], a member of the aromatic amino acid group [24], and the largest of the twenty amino acids. Essential amino acids cannot be synthesized in the human body and are thus supplied through diet for the growth and development of the human body [23]. Trp has comparatively small tissue storage in humans [25], and the overall Trp content in the body is the least of all amino acids, even though very small quantities are required for general healthy nutrition. The most common sources of Trp are cheese, banana, chocolate, milk, eggs, pumpkin, sesame seeds, tofu, peanut, oatmeal, turkey, dried prunes, chicken, bread, peanuts, and tuna fish [23]. In general, essential amino acids are produced by plants and microbes, and when present in animal diets, they are mostly obtained from plants [24].

Trp is primarily (90–95 %) bound to albumin in plasma following ingestion from dietary protein digestion, with the rest 5–10 % unbound and so are rapidly available for tissue uptake and metabolism [26]. The primary function of Trp in the human body is its involvement in protein synthesis. Only the l-isomer of amino acids, including L-Trp, is employed in protein synthesis. L-Trp is capable of crossing the blood-brain barrier [22]. Due to the lower amounts of Trp in the human body, it is assumed to be a very important component in protein synthesis. The body's average Trp protein level is 1.2 g per 100 g of protein, which is much lower than other essential amino acids like lysine (7.6 g), leucine (7.1 g), and threonine (4 g) [27]. Apart from protein synthesis, Trp is also the precursor for two important metabolic pathways, the serotonin pathway and the kynurenine pathway [22].

The kynurenine pathway (KP) accounts for 90 % of Trp catabolism and produces key metabolites, most importantly quinolinic acids and kynurenine. The first step of KP under the action of the enzyme indoleamine-2,3-dioxygenase (IDO) or Trp-2,3-dioxygenase (TDO) is the rate-limiting step of this pathway leads to the production of N-formyl Kynurenine (NFK) [27]. These two enzymes differ in their function, structure, cofactor requirement, tissue localization, and substrate specificity. TDO is mostly found in the liver, whereas IDO is found in a variety of tissues [27]. IDO is expressed in a variety of immune cells, including monocytes, macrophages, dendritic cells (DCs), and microglia, as well as fibroblasts, endothelial cells, epithelial cells, and smooth muscle cells [28]. Interferon (IFN) is one of the primary stimuli that stimulate IDO production at the transcriptional level (Mándi et al., 2012). NKF is hydrolyzed to kynurenine (Kyn) by the enzyme NFK formidase. Kyn is then broken down into a number of metabolites by various enzymes. Quinoline is generated in the final step in which nicotinamide adenine dinucleotide (NAD) is generated [29]. Multiple clinical disorders, including AIDS-related dementia, multiple sclerosis, and ischemic brain damage, can be caused by the accumulation of these and other metabolites in this pathway [30].

The serotonin pathway (about 1–2% of Trp breakdown) is driven by Trp hydroxylase (TPH). It results in the production of the neurotransmitter serotonin also known as 5-hydroxytryptamine (5-HT), which is a precursor to melatonin [29]. In the gastrointestinal tract, 5-HT controls gastrointestinal motility, vascular tone, primary hemostasis, and cell-mediated immunity [31]. Indole metabolites are also produced in the gut by microbiota which is important in intestinal immunity. Mice lacking dietary Trp exhibited impaired intestinal immunity and microbial dysbiosis [32]. In the central nervous system, 5-HT controls mood, food intake, anxiety, and sleep. Melatonin on the other hand aids in the maintenance of the body's circadian rhythm, namely the wake-sleep cycle, as well as the body temperature cycles [33]. It can also act as a scavenger for free radicals. Additionally, melatonin regulates blood pressure and autonomic cardiovascular control, the immune system, and a variety of physiological activities such as retinal functioning, and antioxidant effects that protect the brain from oxidative stress [34]. Melatonin influences plants' responses to both biotic and abiotic stress [35]. As a result, Trp availability is a crucial element in protein biosynthesis regulation. This might be one of the reasons why the immune system uses Trp deficiency to prevent infections and cancer cells from multiplying. Furthermore, kynurenine promotes the formation of regulatory T cells, and several Trp catabolites, such as 3-hydroxyanthranilic and quinolinic acid, have been demonstrated to induce Th1 cells towards apoptosis [36].

*T. brucei* and *Leishmania* spp are known to be auxotrophic for Trp and other nitrogen-containing primary metabolites such as leucine, isoleucine, valine, and purines [37]. The importance of Trp and its metabolites has been shown to regulate the growth of kinetoplastids in the host [38]. This was evidenced from the presence of Trp-like epitopes which were found in HAT and the depletion of L-Trp affected serotonin metabolism and serotonergic functions [39]. In addition, it has been observed that amino acid metabolites like kynurenine which is produced by Indoleamine 2,3-dioxigenase (IDO) from Trp regulate parasite growth which may contribute to the pathophysiology of kinetoplastids diseases. IDO is a rate-limiting enzyme of Trp catabolism that is activated by inflammatory cytokines and is involved in the prevention of intracellular pathogen growth, as well as immunomodulation. Infection with *T. cruzi* has been shown to result in the systemic activation of IDO. The in vivo inhibition of IDO activity has been reported to increase the parasite number substantially in trypanosomiasis [39] and infection-related pathologies while reducing infection resistance [40]. Moreover, to create novel therapies for kinetoplastid infection, various research has investigated how metabolic modulators affect immunological responses. Knubel et al. observed that mice infected with *T. cruzi* treated with 3-hydroxykynurenine in the acute disease stage showed reduction in inflammation and fibrosis in the heart, chronic-stage electrocardiogram alterations, and decreased number of parasites [41]. Contrarily, inhibiting IDO, the enzyme responsible to produce these metabolites, markedly decreased the size of cutaneous leishmaniasis lesions and the parasite load [42]. A metabolomic study involving cerebrospinal fluid (CSF) and plasma from Angolan patients showed many indicators that differed according to disease stage in HAT. The CSF samples of stage 2 HAT showed a rise in neopterin and hydroxyTrp levels, a slight rise in kynurenine, and a decrease in Trp compared to stage 1 HAT [43].

These studies indicate the potential therapeutic activity of Trp, its metabolites and corresponding enzymes that warrant further research to identify new treatment strategies for diseases caused by kinetoplastids.

## 5 Structure and biosynthesis of tryptophan derivatives

Researchers have been interested in chemical modifications of amino acids with indoles and their related compounds to discover new derivatives that may have a therapeutic effect against many diseases by modifying their chemical structure. Amino acid derivatives that have been chemically produced have a wide assortment of chemical structures and are mostly used for producing pharmaceutically active products. A substance derived from Trp by reaction with either the amino or carboxyl groups, or by replacing any hydrogen in Trp with a heteroatom is known as a Trp derivative. These derivatives display a wide range of biological activities [44]. Some common Trp derivatives include 5-Benzyloxy-DL-Trp, 5-Hydroxy-L-Trp (5-HTP), *N*-Acetyl tryptamine, 5-Methyl-DL-Trp, 5-hydroxytryptamine (serotonin) and melatonin [12,45,46]. Most of these derivatives are found as intermediate metabolites in biochemical pathways. Understanding their biosynthesis is essential in assisting chemists in developing simple and effective synthetic pathways to these natural compounds, as well as exploring their pharmacological usage as most of them are important curative agents [27].

**Fig. 4:**
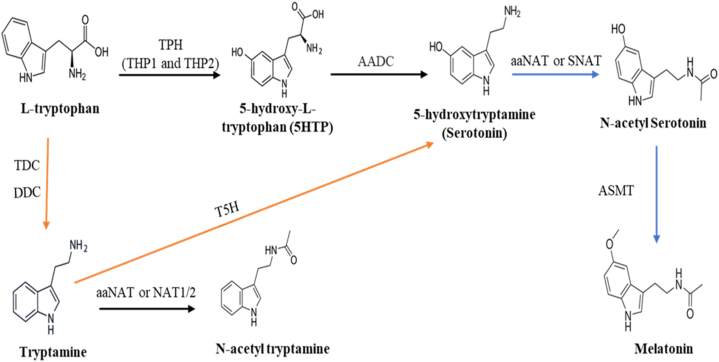
Schematic diagram showing the biosynthesis of Trp derivatives in animals and plants. In animals L-Trp is converted to 5HTP which is then converted to serotonin and melatonin in a series of reactions [48]. In plant however, serotonin is produced from the hydroxylation of tryptamine and the production of melatonin from serotonin occurs in a similar manner as in animals [49]. Black arrow – occurs only in animals, orange arrow – occurs only in plants, Blue Arrow – occurs in both plants and animals.

In humans, 5-HTP is a key intermediate responsible for the biogenesis of intracerebral amine-type hormones, such as serotonin, 5-methoxytryptamine, and N-acetyl-5-methoxytryptamine. Over the last 30 years, 5HTP has been utilized to treat various serotonin-related disorders, including depression, chronic migraines, insomnia, etc. [47]. Production of 5-HTP by biological means is advantageous due to its short production cycle, continuous production, and mild reaction conditions. In the biosynthesis of 5-HTP, L-Trp is used as a substrate where it is converted to 5-HTP in a rate-limiting step during the production of serotonin catalyzed by the enzyme Trp hydroxylase (TPH) (Fig. 4). TPH is a monooxygenase that catalyzes the catalytic process by using Trp and oxygen as substrates and tetrahydrobiopterin (BH4) and Fe^2+^ as cofactors. There are two forms of THP in vertebrates: THP1 and THP2. TPH1 is involved in the synthesis of serotonin in peripheral tissues and is found mostly in enterochromaffin cells of the gut and the pineal gland whiles TPH2 is found in myenteric neurons in the gut and raphe nuclei neurons in the brain stem, but not in other peripheral organs [48]. 5-HTP is subsequently decarboxylated by the enzyme aromatic amino acid decarboxylase (AADC) to produce serotonin (Fig. 4). Serotonin is produced in both plants and animals. In plants, it is produced from the hydroxylation of tryptamine by the enzyme tryptamine-5-hydroxylase (T5H) (Fig. 4).

Melatonin, also known as 5-Methoxy-N-Acetyltryptamine is another Trp derivative that is synthesized in plants, animals, and microorganisms. It is primarily produced in the roots and leaves in plants, specifically in the mitochondria and chloroplasts while in invertebrates, it is mainly produced in the pineal gland and later released into the bloodstream [50]. The biosynthesis of melatonin is different in various organisms. In animals, biosynthesis occurs in four steps where the first two steps are found in the biosynthesis of serotonin. Serotonin will in this case serve as a precursor for the production of melatonin. Acetyl CoA is produced under the action of aralkylamine N-acetyltransferase (aaNAT) or serotonin N-acetyltransferase (SNAT) where serotonin is converted to N-acetyl serotonin, which is subsequently converted to melatonin by the enzyme N-acetyl-serotonin methyltransferase (ASMT). However, in plants, the biosynthesis takes place in five steps where L-Trp is first decarboxylated and converted to tryptamine by the enzyme TDC. Tryptamine is further converted to serotonin by tryptamine-5-hydroxylase (T5H). Through acetylation, serotonin is converted to N-acetyl-serotonin which is then converted to melatonin by methylation (Fig. 4) [49]. N-acetyl tryptamine is also another derivative of Trp which is synthesized by first decarboxylating L-Trp to tryptamine by the enzyme aromatic amino acid decarboxylase (DDC). Tryptamine is then acetylated to N-acetyl tryptamine by the enzymes arylalkylamine N-acetyltransferase and arylamine N-acetyltransferase 1 (NAT1) and 2 (NAT2) (Fig. 4) [45].

The biosynthesis of the aforementioned Trp derivatives in microorganisms has also gained traction in biotechnology. In 1978, Iriuchijima and Tsuchihashi synthesized 5-HTP from N-acetyl-glutamic-γ-semialdehyde, which was done enzymatically from the cell homogenate of the bacterium, *Corynebacterium glutamicum.* The synthesis involved the preparation of the precursor (N-acetyl-glutamic-γ-semialdehyde) and a series of steps that yielded the production of 5-HTP [51]. Recently, an artificial pathway for the biosynthesis of 5-HTP and serotonin in *E. coli* was developed [47]. Compared to *E. coli*, Zhang et al. also found that *S. cerevisiae* can tolerate harsher fermentation conditions and does not have a phage contamination issue. Their findings discovered two metabolic pathways in *S. cerevisiae* that can lead to the production of 5-HTP [52].

Additionally, separate branches of Trp metabolism also create a number of physiologically relevant indole derivatives. Tryptophol, also known as indole-3-ethanol (indole–CH_2_–CH_2_OH), is a common aromatic alcohol. Its biosynthesis route (also known as the ‘Ehrlich pathway’ after its discoverer Felix Ehrlich) starts with Trp deamination to 3-indole pyruvate, decarboxylation to indole acetaldehyde, and final reduction to the alcohol by-alcohol dehydrogenase [24]. Tryptophol is mostly generated by plants and lower eukaryotes such as yeast, fungus, marine sponges, and the unicellular protozoan parasite *Trypanosoma brucei*, which causes the deadly African sleeping sickness [24].

**Fig. 5:**
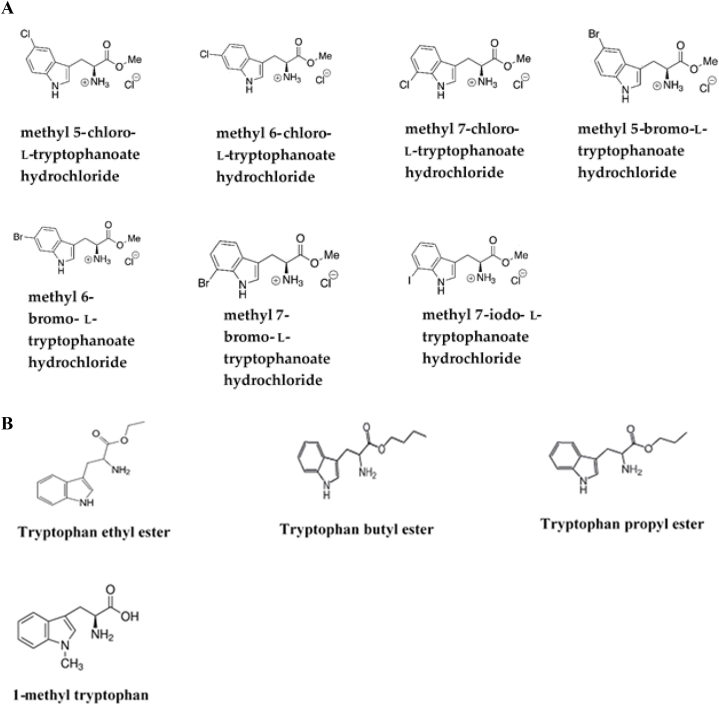
A-Halogenated Trp esters with potential chemotherapeutic effects on transamination mechanisms in bloodstream forms of *T. brucei* [37]. B- Esterified and methylated Trp derivatives [55].

The introduction of functional groups such as halogen alters the characteristics of Trp, changing its fluorescence and lipophilicity while giving a chemically derivatized, selective handle that may be changed even under moderate aqueous conditions [53]. The indole aromatic heterocyclic backbone found in 5HTP, and serotonin is a useful chemical framework with several applications in medicinal chemistry and drug development. Smith et al. (2014) investigated modified Trp synthase enzymes capable of generating a variety of halogenated Trp derivatives from halo-indole and serine [54]. Halogenated Trp esters (Fig. 5A), Trp alkaloids, and 1-methyl-L-Trp, Trp methyl/ethyl/butyl esters (Fig. 5B), and other Trp derivatives are gaining interest due to their biological and medicinal advantages.

## 6 Pharmacological and medicinal significance of tryptophan derivatives

The design and synthesis of unnatural amino acids have sparked attention due to the discovery of possible medicinal compounds based upon peptide structure. As a result, several amino acid chemical changes have been documented. For more than a century, scientists have been interested in the indole ring and its derivatives. This is partly because indole moieties may be found in a wide range of naturally occurring chemicals with a wide range of physiological properties [12]. Due to its basic function as a precursor to numerous bioactive metabolites, Trp has been implicated in a multitude of illnesses and disorders, owing to its significance in the enhancement of health and nutrition, as well as in diagnostic tools and therapeutic agents. Therapeutic methods based upon Trp chemical characteristics are currently being developed. Even though dietary intake of Trp would rarely contribute significantly to Trp availability, supplementation of exogenous Trp has been the topic of several clinical studies and homeopathic applications. There are a plethora of diseases that Trp and its derivatives have been clinically used to treat viz. pain, sleeplessness, depression, chronic tiredness, and bulimia hyperactivity disorder just to mention a few. Trp is commonly used over-the-counter to promote better sleep, relieve anxiety and depression, increase emotional well-being, treat eating disorders, and aid in the management of pain tolerance [22].

Serotonin and 5HTP possess an indole aromatic heterocyclic backbone, which provides a plethora of medicinal chemistry and drug discovery possibilities. 5HTP is commercially manufactured from the seeds of *Griffonia simplicifolia*, an African plant [47]. It is an over-the-counter drug in countries such as the USA, Canada, and the Netherlands. It is also used in the UK as a food supplement for depressed individuals and also to improve the sleep cycle of people suffering from insomnia. Several clinical trials have confirmed the antidepressant activity of 5HTP [56]. In addition, 5-HTP may help to prevent migraines, as well as lower the incidence and severity of migraines since serotonin has been shown to play an important role in the pathophysiology of migraines. For example, as far back as 1986, work done by Titus and colleagues reported that 5HTP can be used as a treatment for migraine attacks [57]. Another study was done for four months and also concluded the reduction of migraine when treated with 5HTP [58]. For disorders involving serotonin depletion or malfunction, the serotonin pathway has been identified as a possible therapeutic target. As a result, 5HTP and serotonin might be employed as building blocks for pharmacologically active substances.

Melatonin has also been shown to have therapeutic effects in a variety of conditions, including cancer, cardiovascular disease, and mental problems [34]. Melatonin is a naturally occurring oncostatic (halts the spread of cancer) agent and its effects have been implicated in several cancer types including breast cancer [59,60], colon cancer [61], prostate cancer [62,63], human uveal melanoma [64], ovarian cancer [65]. It has been suggested that these effects are due to the anti-oxidative properties of melatonin [66] because carcinogenesis is triggered, promoted, and progressed by oxidative stress [67]. In addition, melatonin has been shown to reduce blood pressure in hypertensive individuals [68,69]. Due to its ability to cross both the blood-brain and placenta barriers, melatonin can be given antenatally to lessen or avoid the impact of brain injuries in premature newborns [70]. Melatonin has recently been examined as a possible adjuvant medication for reducing the effects of COVID-19 [71]. Melatonin has also been shown to have therapeutic promise in neurological diseases such as Alzheimer's disease, Parkinson's disease, and Huntington's disease in recent studies [72].

Natural products have long been significant in medication development, either as stand-alone molecules or as inspiration for synthetic drugs. The chemical synthesis of various Trp derivatives as a therapeutic agent is gaining lots of attention these days. Many non-ribosomal peptide antibiotics use Trp, and its derivatives as biosynthetic precursors and these peptides have been shown to have a diverse range of biological functions. For example, the argyrin family which contains (*S*)-4-methoxy-Trp residue has been shown to possess antibacterial and antifungal activity [73]. Furthermore, 1-methyl-L-Trp (1-MT), an inhibitor of the enzyme, Indoleamine 2,3-dioxygenase (IDO), has been demonstrated to increase apoptosis in hepatic stellate cells, and its co-treatment with interferon-***γ*** reduced cardiac fibrosis through apoptosis [74]. Trp alkaloids such as quinolines, ergot alkaloids and *β*-carbolines are also gaining traction due to their therapeutic properties against several pathogens [75]. The anticancer properties of Trp derivatives can also be seen in methyl-Trp derivatives and IDO inhibitors which are potential medicines for treating tumor development and metastasis formation due to IDO-related immune tolerance for cancer antigens. The anti-cancer activity of the compounds was observed and reported that these derivatives reduced the spread and growth of breast cancer cells [76].

Trp may also exhibit extensive medicinal properties. Trp-containing compounds have been investigated as potential therapeutics for protein aggregation in neurodegenerative diseases [77]. Alternative strategies for treating CNS diseases include focusing on the kynurenine shunt and its modulation, which acts at the heart of the Trp fate balance: since kynurenine derivatives are linked to both N-Methyl-d-aspartic acid (NMDA) agonism and antagonism, as well as nicotinic acid pathways, their metabolism can be studied for treating cognitive deficits, dementia, and other severe neuropsychiatric conditions [77]. Moreover, Trp and its derivatives have been used as biomarkers for the diagnosis of several ailments. Gakamsky et al. (2017) reported that Trp and its derivatives can be used as biomarkers for cataract diagnosis at the molecular level [78]. Also, it has been reported that serum concentration of Trp can serve as the prognostic marker in patients with diabetic nephropathy [79]. Several enzymes in the Trp-kynurenine pathway may also serve as possible therapeutic targets for the treatment of metabolic, neurological, and psychiatric diseases, as well as cancer [36].

## 7 Anti-kinetoplastids potential of tryptophan derivatives

Diseases caused by kinetoplastids including African Trypanosomiasis, Chagas Disease and Leishmaniasis are claiming millions of lives every single year particularly in developing nations. Vector control and chemotherapy are being used for controlling these diseases. Existing treatments have become ineffective due to their toxicity and severe side effects posed to individuals with the disease and the increasing emergence of resistance. This has necessitated the need for effective drugs with little or side effects. Natural substances are gaining traction for treating parasite illnesses. Natural products provide an alternate source of unknown biologically active chemicals that might be used as precursors for the development of effective treatment drugs against parasitic diseases. Recent attempts to decode the structural and biological properties of natural products with anti-kinetoplastid activity have generated compounds with potential anti-kinetoplastid therapeutic effect. Due to its important function as a precursor to various bioactive metabolites, as well as its usefulness in the enhancement of health and nutrition, diagnostics, and therapies, Trp has been implicated in a wide range of illnesses and disorders.

An essential part of the development of kinetoplastids is the control of Trp metabolism making the exploration of this area a potential target for developing effective drugs against kinetoplastids. The oxidative conversion of Trp to kynurenine molecules is a key metabolic route that has received little attention in the context of trypanosomiasis. This route accounts for the bulk of non-protein Trp metabolism in most tissues, and various components of the process have considerable impacts on neuronal activity in the CNS. Amino acid absorption is critical for trypanosomatid parasites, and *T. brucei and Leishmania* are auxotrophic for several amino acids. As a result, amino acid metabolism has been identified as a promising target for the treatment of trypanosomatid-related disease, with the potential for high parasite specificity. The kynurenine pathway of Trp metabolism has been found to be activated and related to central nervous system inflammation in rat models of human African trypanosomiasis [80]. This raises the possibility that the comprehensive exploration and investigation of Trp metabolism and its associated impact on HAT could potentially shed light on its role in the disease pathogenesis and ultimately may contribute to discovering potential drug targets.

Melatonin, a naturally occurring Trp derivative and an important antioxidant agent, has been proven to confer protection against Chagas diseases (caused by *T. cruzi*) by inducing the production of proinflammatory cytokines [[81], [82], [83]]. The incubation of epimastigotes with exogenous melatonin did not influence parasite development, however, it did considerably diminish metacyclic transformation after 7–8 days of treatment as reported by Ref. [84]. IDO, an interferon-inducible enzyme that catalyzes the first and rate-limiting step of Trp degradation via the l-kynurenine pathway, is essential for host immunity to parasite infection. In mice infected with *T. cruzi*, IDO was found up-regulated and its inhibition led to an increase in the parasite load [41]. Furthermore, unlike other intracellular pathogens that are sensitive to Trp depletion, *T. cruzi* is sensitive to 3-hydroxykynurenine (3-HK), and therapeutic administration of 3-HK during the acute phase of infection reduces parasite load in blood and target tissues and improves the survival of lethally infected mice. Furthermore, 3-HK was active against trypomastigotes and amastigotes at doses that were not cytotoxic to mammalian cells [41]. According to these findings, we can infer that Trp derivatives have a great influence on the growth of kinetoplastids though further experiments is needed to make a definite conclusion. Notwithstanding, they are potential agents with anti-kinetoplastid activity.

Furthermore, *T. brucei* engages in the extensive metabolism of amino acids for diverse cellular activities throughout the various morphologies of its life cycle, in addition to their functional usage in protein synthesis. One of the most crucial metabolic processes is the transamination of aromatic amino acids. A recent paper investigated and revealed the potency of halogenated Trp derivatives which disrupted the mechanism of transamination in the bloodstream form of *T. brucei*. Seven free acids of Trp derivatives in addition to the natural L-Trp and their corresponding methyl esters were investigated for their antiparasitic potency against *T. brucei* bloodstream form trypomastigotes, *T. brucei* procyclic form trypomastigotes, *T. cruzi* epimastigotes, *L. major* promastigotes, and HeLa cells [37]. It was realized that methyl esterification of the compounds increased the trypanocidal activity of the compounds with the exception of the natural L-Trp. Moreover, none of the compounds investigated were cytotoxic to the HeLa cells. These compounds might thus be utilized to help future research into the functional relevance of *T. brucei* aromatic amino acids transamination processes.

Moreover, Mohareb and colleagues (2009), explored the use of L-Trp as a starting material to form compounds with potential biological activities. The researchers synthesized a plethora of indole derivatives containing heterocyclic moiety which have been found to contain thiophene, pyarazole and pyridine which are potentially therapeutic agents. One of key compounds they synthesized is the methyl imino (acetonitrilocarbamido)-3-indolopropanoate which they concluded to be an interesting compound to chemically modify to generate indole derivatives with potential biological activity. For example, the above-mentioned compound was reacted with hydrazine hydrate to give an indole derivative called pyrozolTrp methyl ester derivative [12]. Though not explicitly stated by the authors the exact biological activity of this compound, it can be inferred that the chemical modifications of this compounds can lead to the formation of indole derivatives which can be explored against parasitic infections such as diseases caused by kinetoplastids.

Dofuor et al. (2021) investigated the antitrypanosomal activity of *Bidens pilosa* plant fractions, finding out that the methanol fraction (BPFM) exhibited the strongest effect on trypanosomes. They identified two compounds butyl and propyl esters of Trp after isolating and analyzing the fractions to explore the chemicals responsible for this trypanocidal activity. When compared to the conventional antitrypanosomal medication diminazene, cell viability studies on these Trp esters extracted further verified their antitrypanosomal potencies. Two of the compounds, Trp butyl ester, and Trp propyl ester had strong antitrypanosomal actions on *T. brucei,* with IC_50_ values of 0.66 and 1.46 μg/ml, respectively, indicating that these compounds contributed considerably to the *T. brucei* growth suppression reported for fraction BPFM. The compounds demonstrated reasonably non-cytotoxic effects on normal macrophages RAW 264.7 cell lines indicating how the therapeutic value of these compounds will not be compromised upon use [55]. Due to the broad-spectrum effectiveness of anti-protozoal agents, these compounds might have activity against other protozoans. These compounds, therefore, warrant further research to be used as therapeutic agents in treating trypanosomiasis.

Overall, Trp and its derivatives are potential therapeutic agents to be explored against kinetoplastids looking at the urgency and necessity in developing new and effective drugs to overcome the issues of toxicity, side effect and resistance to the existing treatments for kinetoplastid-related diseases.

## 8 Conclusion and future prospects

A wide variety of different compounds obtained from animals, plants, microorganisms, and marine species have been employed to treat human ailments throughout the last century [85]. Well-known drugs obtained from natural sources include the penicillin antibiotic, artemisinin antimalarial, and morphine analgesic. Even though natural products have a demonstrated record of structural diversity and success in drug development, they have a number of challenges [86]. As with a number of isolated natural compounds, Trp derivatives may have certain physicochemical limits that would need pharmacological modification to achieve the desired chemotherapeutic characteristics [86]. In addition, extract libraries of natural product screens may not be suitable for traditional target-based assays [87]. Furthermore, despite their antiparasitic efficacy, the mechanism of action of Trp derivatives continues to receive little attention. Moreover, there is the risk of poor solubility and chemical instability [86]. Also, the cytotoxicity of Trp analogs at high concentrations in mammalian cells can be a problem [88]. Despite these challenges, there is sufficient evidence to support Trp derivatives as potential anti-kinetoplastids. Additional investigation into the toxicity, target identification, physicochemical properties, and in vivo mode of action of anti-kinetoplastids will be required to advance their discovery and development into commercially available drugs.

## Data availability statement

Data included in article/supp. material/referenced in article

## Funding

This study was not supported by grant from any institution or government.

## CRediT authorship contribution statement

**Ewura-Esi Manful:** Writing – review & editing, Writing – original draft, Visualization, Validation, Methodology, Investigation, Formal analysis, Data curation. **Aboagye Kwarteng Dofuor:** Writing – review & editing, Writing – original draft, Visualization, Validation, Supervision, Methodology, Investigation, Formal analysis, Data curation, Conceptualization. **Theresa Manful Gwira:** Writing – review & editing, Writing – original draft, Supervision.

## Declaration of competing interest

The authors declare that there is no known competing financial interests or personal relationships that could have appeared to influence the work reported in this paper.
